# Associations Between Findings From Myelin Water Imaging and Cognitive Performance Among Individuals With Multiple Sclerosis

**DOI:** 10.1001/jamanetworkopen.2020.14220

**Published:** 2020-09-29

**Authors:** Shawna Abel, Irene Vavasour, Lisa Eunyoung Lee, Poljanka Johnson, Stephen Ristow, Nathalie Ackermans, Jillian Chan, Helen Cross, Cornelia Laule, Adam Dvorak, Alice Schabas, Enedino Hernández-Torres, Roger Tam, Annie J. Kuan, Sarah A. Morrow, Jeffrey Wilken, Alexander Rauscher, Virender Bhan, Ana-Luiza Sayao, Virginia Devonshire, David K. B. Li, Robert Carruthers, Anthony Traboulsee, Shannon H. Kolind

**Affiliations:** 1 Department of Medicine (Neurology), The University of British Columbia, Vancouver, British Columbia, Canada; 2 Department of Radiology, The University of British Columbia, Vancouver, British Columbia, Canada; 3 Department of Pathology & Laboratory Medicine, The University of British Columbia, Vancouver, British Columbia, Canada; 4 Department of Physics & Astronomy, The University of British Columbia, Vancouver, British Columbia, Canada; 5 International Collaboration on Repair Discoveries, The University of British Columbia, Vancouver, British Columbia, Canada; 6 Department of Psychiatry, The University of British Columbia, Vancouver, British Columbia, Canada; 7 Department of Clinical Neurological Sciences, Western University, London, Ontario, Canada; 8 Department of Neurology, Georgetown University Hospital, Washington, DC; 9 Washington Neuropsychology Research Group LLC, Fairfax, Virginia; 10 Department of Pediatrics, The University of British Columbia, Vancouver, British Columbia, Canada

## Abstract

**Importance:**

Cognitive impairment is a debilitating symptom of multiple sclerosis (MS) that affects up to 70% of patients. An improved understanding of the underlying pathology of MS-related cognitive impairment would provide considerable benefit to patients and clinicians.

**Objective:**

To determine whether there is an association between myelin damage in tissue that appears completely normal on standard clinical imaging, but can be detected by myelin water imaging (MWI), with cognitive performance in MS.

**Design, Setting, and Participants:**

In this cross-sectional study, participants with MS and controls underwent cognitive testing and magnetic resonance imaging (MRI) from August 23, 2017, to February 20, 2019. Participants were recruited through the University of British Columbia Hospital MS clinic and via online recruitment advertisements on local health authority websites. Cognitive testing was performed in the MS clinic, and MRI was performed at the adjacent academic research neuroimaging center. Seventy-three participants with clinically definite MS fulfilling the 2017 revised McDonald criteria for diagnosis and 22 age-, sex-, and education-matched healthy volunteers without neurological disease were included in the study. Data analysis was performed from March to November 2019.

**Exposures:**

MWI was performed at 3 T with a 48-echo, 3-dimensional, gradient and spin-echo (GRASE) sequence. Cognitive testing was performed with assessments drawn from cognitive batteries validated for use in MS.

**Main Outcomes and Measures:**

The association between myelin water measures, a measurement of the T2 relaxation signal from water in the myelin bilayers providing a specific marker for myelin, and cognitive test scores was assessed using Pearson correlation. Three white matter regions of interest—the cingulum, superior longitudinal fasciculus (SLF), and corpus callosum—were selected a priori according to their known involvement in MS-related cognitive impairment.

**Results:**

For the 95 total participants, the mean (SD) age was 49.33 (11.44) years. The mean (SD) age was 50.2 (10.7) years for the 73 participants with MS and 46.4 (13.5) for the 22 controls. Forty-eight participants with MS (66%) and 14 controls (64%) were women. The mean (SD) years of education were 14.7 (2.2) for patients and 15.8 (2.5) years for controls. In MS, significant associations were observed between myelin water measures and scores on the Symbol Digit Modalities Test (SLF, *r* = −0.490; 95% CI, −0.697 to −0.284; *P* < .001; corpus callosum, *r* = −0.471; 95% CI, −0.680 to −0.262; *P* < .001; and cingulum, *r* = −0.419; 95% CI, −0.634 to −0.205; *P* < .001), Selective Reminding Test (SLF, *r* = −0.444; 95% CI, −0.660 to −0.217; *P* < .001; corpus callosum, *r* = −0.411; 95% CI, −0.630 to −0.181; *P* = .001; and cingulum, *r* = −0.361; 95% CI, −0.602 to −0.130; *P* = .003), and Controlled Oral Word Association Test (SLF, *r* = −0.317; 95% CI, −0.549 to −0.078; *P* = .01; and cingulum, *r* = −0.335; 95% CI, −0.658 to −0.113; *P* = .006). No significant associations were found in controls.

**Conclusions and Relevance:**

This study used MWI to demonstrate that otherwise normal-appearing brain tissue is diffusely damaged in MS, and the findings suggest that myelin water measures are associated with cognitive performance. MWI offers an in vivo biomarker feasible for use in clinical trials investigating cognition, providing a means for monitoring changes in myelination and its association with symptom worsening or improvement.

## Key Points

**Question:**

Is myelin damage in normal-appearing white matter associated with cognitive impairment in participants with multiple sclerosis (MS)?

**Findings:**

In this cross-sectional study of 73 participants with MS and 22 age-, sex-, and education-matched healthy controls, significant associations were observed in participants with MS between a quantitative neuroimaging measure of myelin and performance on cognitive tests validated for MS. No significant associations were found between myelin measures and cognitive performance in controls.

**Meaning:**

These findings suggest that myelin damage that is completely invisible on standard clinical images but can be measured using myelin water imaging is involved in MS-related cognitive impairment.

## Teaser

This cross-sectional study uses myelin water imaging to examine participants with multiple sclerosis as well as controls to see whether advanced imaging can show myelin damage that does not appear on standard imaging.

## Introduction

Multiple sclerosis (MS) is an inflammatory, neurodegenerative disease of the central nervous system^1^ that affects more than 2 million people globally, rendering it the most prevalent chronic neuroinflammatory disease of the central nervous system worldwide.^2^ Cognitive impairment is a common symptom in MS that presents in up to 70% of patients.^3^ Cognitive symptoms in MS typically manifest as deficits in attention, memory, and processing speed,^3^ with processing speed being most frequently affected.^4^

MS-related cognitive impairment has a substantial impact on quality of life,^5^ including the ability to perform tasks of daily living,^6^ fitness to drive,^7^ and social functioning.^6^ It is also a major contributor to unemployment in patients with MS.^5,8^ Undoubtedly, cognitive impairment presents a major burden to those living with MS, and an improved understanding of its underlying pathology would be of great benefit to patients and clinicians.

MS is characterized by demyelination,^9^ with the radiological hallmark being focal areas of myelin loss, referred to as lesions.^10^ Lesions are visible on conventional T1-weighted and T2-weighted contrast-enhanced magnetic resonance imaging (MRI) and are the mainstay of MS diagnosis and disease monitoring.^11^ However, the association of focal lesion burden with physical and cognitive disability is limited; this is known as the clinicoradiological paradox.^12,13^ One possible cause of this paradox may be because MS pathology extends beyond lesions that are visible on conventional MRI.^14^ Postmortem histopathology studies suggest that normal-appearing white matter (NAWM)—areas that appear normal on standard imaging—is diffusely demyelinated in MS.^15,16^ Investigating the contribution of demyelination within NAWM to clinical outcomes, such as cognition, requires advanced imaging techniques that are quantitative, sensitive, and biologically specific to MS pathology.

Quantitative characterization of myelin in vivo is feasible using myelin water imaging (MWI). MWI separates the MRI signal into contributions from the distinct water pools within a voxel according to the MRI property known as T2 relaxation time. In central nervous system tissue, these water pools generally correspond to (1) a long relaxation time component that arises from cerebrospinal fluid (T2 ≈ 2 seconds), (2) an intermediate component stemming from intracellular and extracellular water (T2 ≈ 60-80 ms), and (3) a short component stemming from water trapped between the myelin bilayers (T2 ≈ 20 ms).^17^ The fraction of MR signal arising from the myelin water divided by the total water signal is the myelin water fraction (MWF). The MWF has been histologically validated as a specific marker for myelin using human tissue^18,19^ and animal models of myelin damage.^20^ At present, MWI is the most direct means of assessing alterations in myelin noninvasively.

Here, we used MWI to investigate the role of myelin damage in NAWM and cognitive function in MS. MWF results are typically described by the mean value within a region of interest (ROI) or by the heterogeneity (variance). To increase sensitivity to disease-associated changes, we characterized the entire MWF distribution by combining both measures as the coefficient of variation (SD / mean), termed the myelin heterogeneity index (MHI), with an increased MHI indicating more myelin damage. To assess cognitive deficits, we used measures from widely used and validated cognitive batteries for MS.^21,22^ Processing speed and attention were measured using the oral version of the Symbol Digit Modalities Test (SDMT), verbal memory with the Selective Reminding Test (SRT), word retrieval with the Controlled Oral Word Association Test (COWAT), and visuospatial memory with the Brief Visuospatial Memory Test Revised (BVMT-R). We selected 3 white matter (WM) ROIs—the cingulum, superior longitudinal fasciculus (SLF), and corpus callosum—a priori on the basis of their known involvement in MS-related cognitive impairment.^23^ We hypothesized that an increased MHI would be associated with worse cognitive performance in MS.

## Methods

### Participants

This study was approved by the University of British Columbia Clinical Research Ethics Board. All participants provided written informed consent. This study followed the Strengthening the Reporting of Observational Studies in Epidemiology (STROBE) reporting guideline.

Participants were recruited through the University of British Columbia Hospital MS clinic and via online recruitment advertisements on local health authority websites. Study appointments took place from August 23, 2017, to February 20, 2019. Seventy-three participants with clinically definite MS fulfilling the 2017 revised McDonald criteria for diagnosis^11^ (38 with relapsing-remitting MS, 12 with primary progressive MS, and 23 with secondary progressive MS) and 22 age-, sex- and, education-matched healthy volunteers without neurological disease were included in the study. All MS phenotypes were recruited to capture varying levels of cognitive disability and MWF values.

### Clinical and Neuropsychological Assessments

To characterize overall disability, participants were examined with the Kurtzke Expanded Disability Status Scale.^24^ To investigate whether our MWI findings were specific to cognition rather than a proxy for physical disability, participants with MS performed the timed 25-foot walk (T25-FW) as a measure of lower limb function and the 9-hole peg test (9-HPT) as a measure of upper limb function. Participants performed a battery of neuropsychological assessments validated for use in MS.

The oral version of the SDMT^21^ was used as a measure of processing speed. One control participant performed the written version with instructions provided by a translator as they were non–English speaking. This test contains a reference key with the numbers 1 through 9 each corresponding to different geometric symbols. The answer key contains only symbols to which the participant must match the corresponding number according to the key. The subject responds orally with the digit associated with the symbol as quickly as possible. The test is scored by tallying the total number of correct responses achieved in 90 seconds.

The SRT^21^ was used to assess verbal memory. The participant is read aloud a list of 12 words that they are asked to repeat back immediately. After they have repeated all words they can remember, the participant is read back only the words they have missed and asked to repeat all 12 words again. This procedure is repeated for 6 rounds. The participant is again asked to repeat all 12 words subsequent to a delay during which they perform other cognitive tests in the battery. The SRT is scored by tallying the total correct recalled words.

Word retrieval was assessed using the COWAT.^21,22^ The participant is given a letter of the alphabet (eg, “F”) and asked to list as many words as they can produce beginning with that letter in 1 minute’s time. The version of the test used in this study included 3-letter prompting categories and an animal category, for which the participant names as many animals as they can recall. The COWAT is scored by tallying the total number of permissible answers; proper names (eg, cities) and variations of the same word (eg, runs, running, ran) are not permitted.

Visuospatial memory was evaluated with the BVMT-R.^22^ The participant is presented with a display of 6 geometric figures for 10 seconds and then asked to reproduce the display by drawing it exactly as it was seen on a blank page. The participant is given 3 opportunities to view and reproduce the display. In the present study, the total recall t-score was used, which is the sum of all valid items generated across learning trials 1 through 3, corrected for age. Lower scores indicate worse performance on all tests.

All participants completed the SDMT at a minimum. However, because of the time constraints of the research appointment, not all participants completed every test. The SRT was completed by 66 patients and 17 controls, 65 patients and 16 controls completed the COWAT, and 63 patients and 15 controls completed the BVMT-R.

### MRI Data Acquisition

MRI scans were conducted on a 3-T scanner (Achieva; Philips Healthcare). Sequences included a 3-dimensional (3D), T1-weighted anatomical scan (whole-brain 3D magnetization-prepared rapid gradient-echo [MPRAGE]; repetition time, 3000 ms; inversion time, 1072 ms; 1 × 1 × 1 mm voxel; 160 slices) for registration and segmentation of WM ROIs and a 48-echo, 3D gradient and spin-echo (GRASE) T2 relaxation sequence with an echo-planar imaging factor of 3 (repetition time, 1073 ms; echo spacing, 8 ms; 20 slices acquired at 1 × 2 × 5 mm reconstructed to 40 slices at 1 × 1 × 2.5 mm) for MWF determination.^25^ A spin-echo, proton density–weighted, T2-weighted scan (repetition time, 2900 ms; echo time, 8.4/80 ms; 0.94 × 0.94 × 3 mm; 54 slices) was also acquired for lesion identification.

### MR Image Registration and Analysis

Voxelwise signal decay curves obtained from the T2 relaxation (GRASE) sequence were modeled by multiple exponential components, with no a priori assumptions about the number of contributing exponentials. Analysis used a regularized, nonnegative, least-squares algorithm with the extended phase graph algorithm and flip angle estimation to correct for stimulated echoes using both in-house software and Matlab version R2013b (MathWorks).^26^ Voxelwise MWF maps were computed as the ratio of the area under the T2 distribution with times of less than 40 ms to the total area under the distribution.^25^

MWF maps were aligned with the anatomical images for each individual by linearly coregistering the 3D T1-weighted images to the first echo of the GRASE scan using FLIRT (with 9 *df*),^27^ which is a linear registration tool and part of the Oxford Centre for Functional MRI of the Brain Software Library (FSL version 5.0.2).^28^ Non–brain parenchyma signal was removed using an automated approach with Brain Extraction Tool, which is part of FSL.^29^ WM masks were generated from the 3D T1-weighted image using the automated brain segmentation algorithm FAST,^30^ which is part of FSL, followed by in-plane erosion using a 2-dimensional kernel and 3 × 3 × 1 box centered on the target voxel to eliminate gray matter and cerebrospinal fluid voxels. The John Hopkins University tract atlas^31^ in the MNI (standard template) space was used to segment 3 WM ROIs (cingulum, SLF, and corpus callosum selected a priori because of their known involvement in MS-related cognitive impairment) using FSL.^23^ The 3D T1-weighted image was nonlinearly warped to MNI space using FNIRT (a nonlinear registration tool), and the ROIs were then transformed onto the MWF map in native space. Each ROI mask was multiplied by the participant’s global WM mask to eliminate gray matter, cerebrospinal fluid, and most of the lesioned tissue, then manually checked and edited as needed. Lesion masks produced by a neurologist were subtracted from the ROI masks to eliminate any remaining lesioned tissue. The MHI was computed for the cingulum, SLF, and corpus callosum NAWM for each individual by dividing the SD of MWF values by the mean MWF.

### Statistical Analysis

All statistical procedures were performed using SPSS Statistics for Mac software version 25.0 (IBM Corp) from March to November 2019. Assumptions of normality were tested with the Shapiro-Wilk test for normality. The assumption of homogeneity of variance was tested with the Levene test of equality of variance. If the assumptions for a parametric test were violated, we proceeded with the appropriate nonparametric test. The Welch *t* test was used to determine whether there was a significant difference in age and years of education between groups. A χ^2^ test was used to determine whether the groups were matched for sex. Associations between MHI and performance on each cognitive test were explored with Pearson correlation. Although all *P* values less than .05 are reported, significance thresholds were set with the Bonferroni correction for the 3 brain regions assessed, with each cognitive domain treated separately (*P* < .016). All tests were 2-sided.

## Results

### Participant Characteristics

There were 95 total participants with a mean (SD) age of 49.33 (11.44) years. Of the 73 participants with MS, the mean (SD) age was 50.2 (10.7) years (range, 26-65 years), 48 (66%) were women, and they had a mean (SD) of 14.7 (2.2) years of education (range, 12-22 years). Participants with MS had a median Kurtzke Expanded Disability Status Scale of 3.5 (range, 1.0-8.5) and median disease duration of 12 (range, 0.3-48) years. Controls had a mean (SD) age of 46.4 (13.5) years (range, 27-65 years), 14 (64%) were women, and they had a mean (SD) of 15.8 (2.2) years of education (range, 12-22 years). Participants with MS and controls did not differ significantly in age, sex, or education. The clinical and demographic characteristics of participants with MS and controls are shown in the Table.

**Table.** Clinical and Demographic Characteristics

Characteristics	Controls (n = 22)	Participants with MS (n = 73)	*P* value
Age, y, mean (range)	46.4 (27-65)	50.2 (26-65)	.24
Female, No. (%)	14 (64)	48 (66)	.85
Education, y, mean (range)	15.8 (12-22)	14.7 (12-22)	.06
Expanded Disability Status Scale score, median (range)	NA	3.5 (1.0-8.5)	NA
Disease duration, median (range), y	NA	12.0 (0.3-48)	NA

Abbreviations: MS, multiple sclerosis; NA, not applicable.

### Symbol Digit Modalities Test

**Figure 1.**
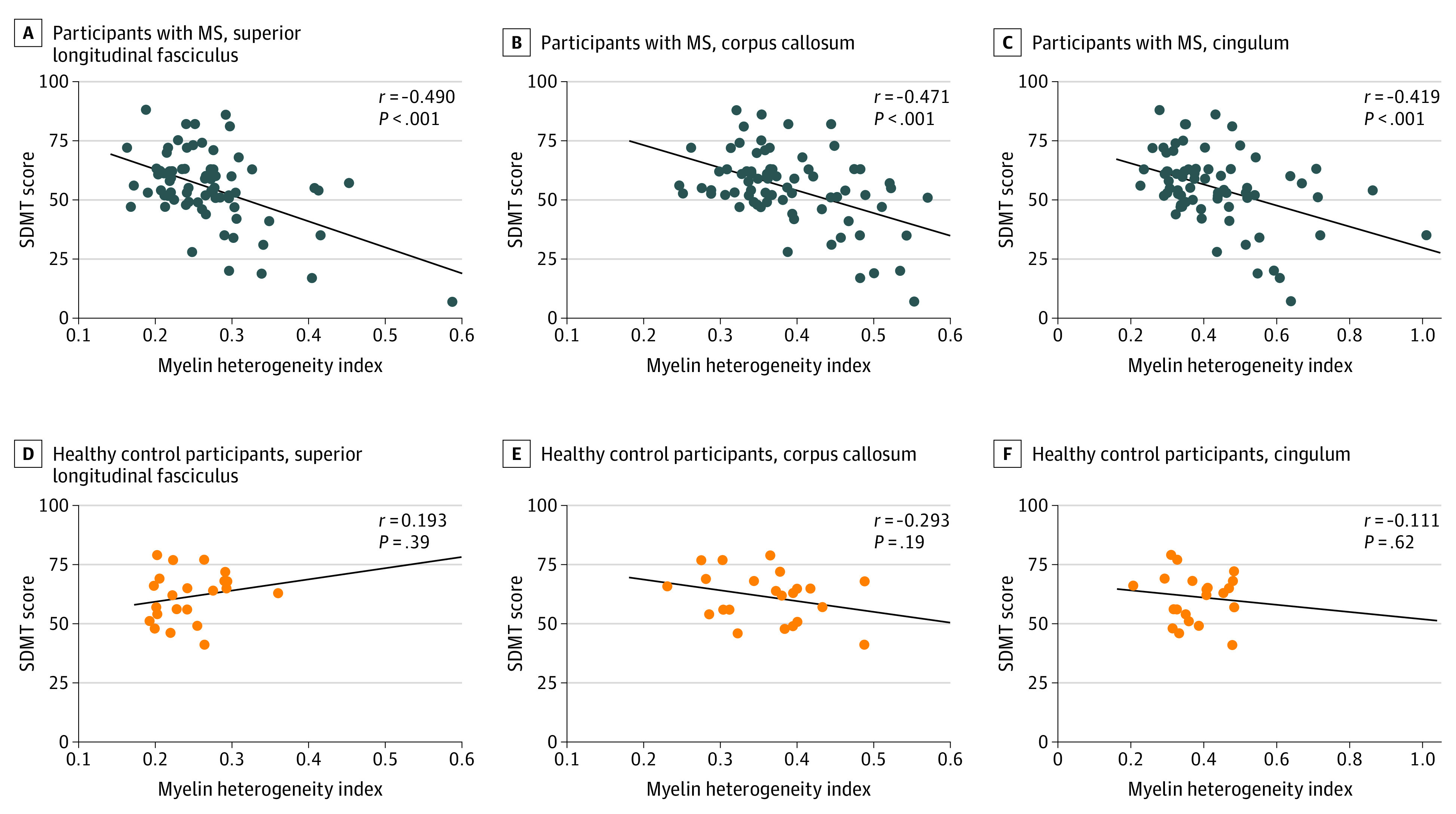
Correlations Between Symbol Digit Modalities Test (SDMT) Performance and Myelin Heterogeneity Index Correlations between the myelin heterogeneity index in normal-appearing white matter and SDMT scores are shown for participants with multiple sclerosis (MS) and controls in 3 regions of interest. Lines denote lines of best fit.

MS patients had a mean SDMT score of 56 (range, 7-88). The mean SDMT score for controls was 62 (range, 41-79). Figure 1 illustrates the correlations between the MHI in NAWM and SDMT scores. In patients with MS, an increased MHI in the SLF (*r* = −0.490; 95% CI, −0.697 to −0.284; *P* < .001), corpus callosum (*r* = −0.471; 95% CI, −0.680 to −0.262; *P* < .001), and cingulum (*r* = −0.419; 95% CI: −0.634 to −0.205; *P* < .001) was associated with worse performance on the SDMT. In controls, MHI was not associated with SDMT performance in any ROI.

### Selective Reminding Test

**Figure 2.**
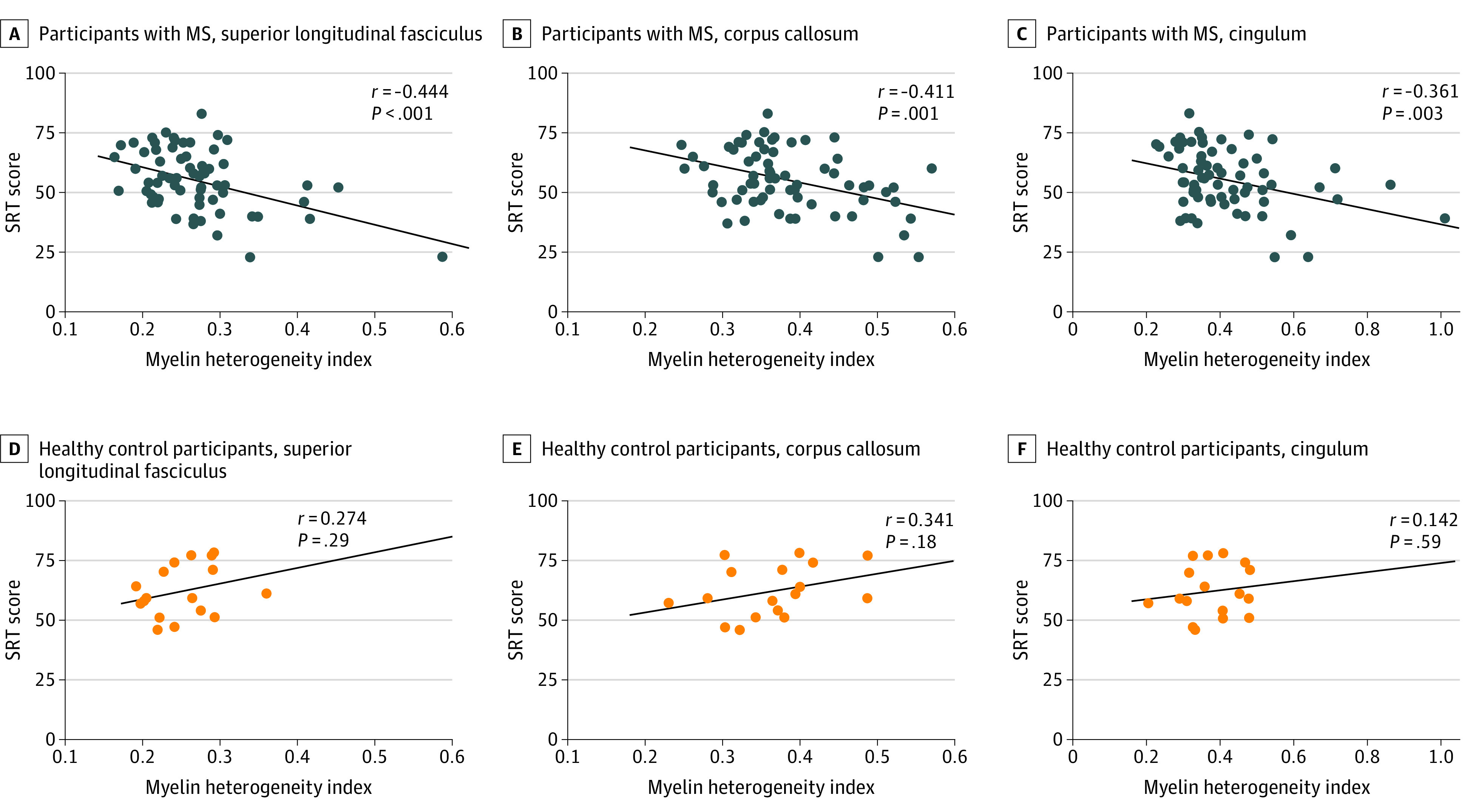
Correlations Between Selective Reminding Test (SRT) Performance and Myelin Heterogeneity Index Correlations between the myelin heterogeneity index in normal-appearing white matter and SRT scores are shown for participants with multiple sclerosis (MS) and controls in 3 regions of interest. Lines denote lines of best fit.

The mean SRT score was 55 (range, 23-83) for participants with MS and 62 (range, 46-78) for controls. Increased MHI in the SLF (*r* = −0.444; 95% CI, −0.660 to −0.217; *P* < .001), corpus callosum (*r* = −0.411; 95% CI, −0.630 to −0.181; *P* = .001), and cingulum (*r* = −0.361; 95% CI, −0.602 to −0.130; *P* = .003) was significantly correlated with worse SRT performance in participants with MS. MHI was not associated with SRT performance in controls (Figure 2).

### Controlled Oral Word Association Test

**Figure 3.**
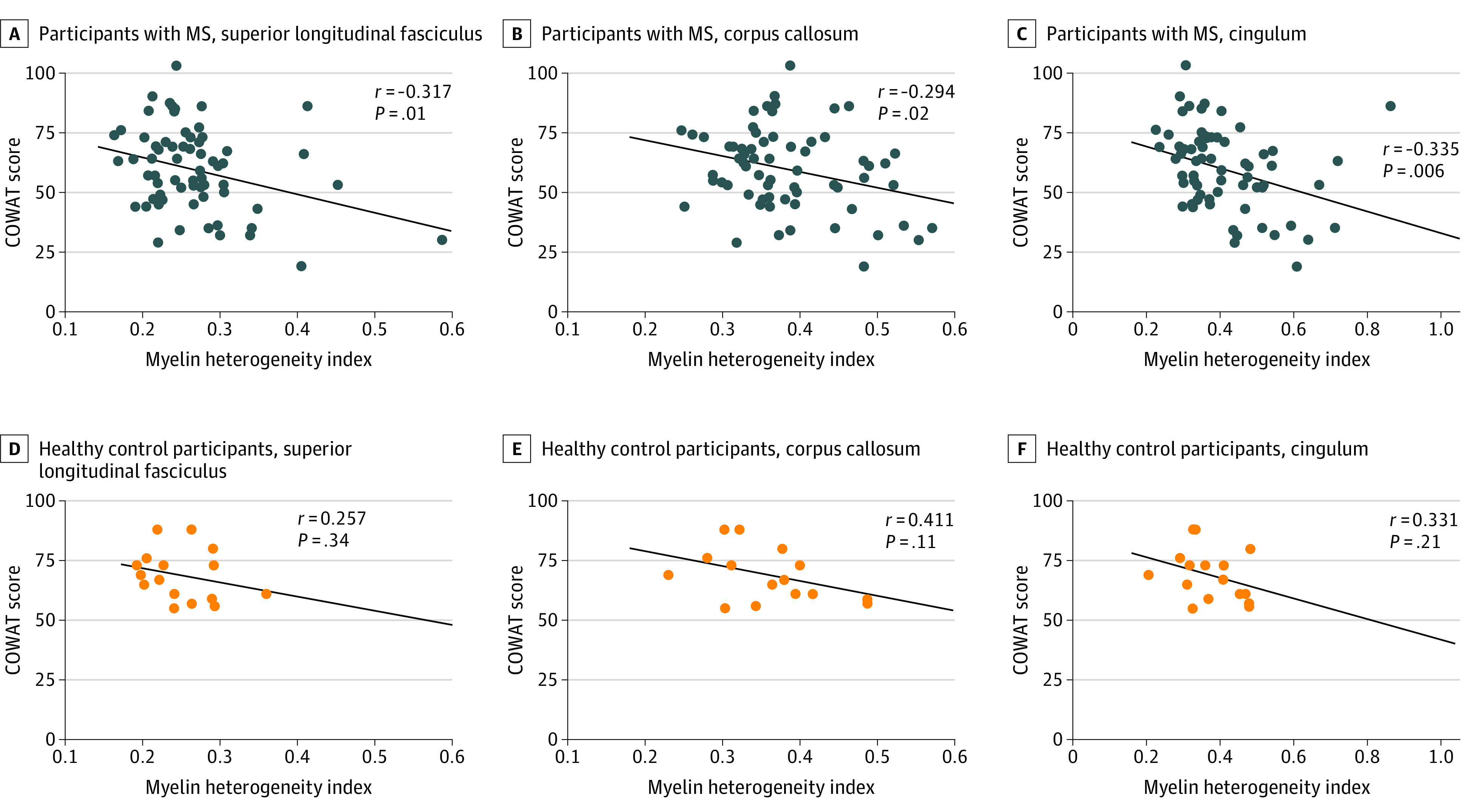
Correlations Between Controlled Oral Word Association Test (COWAT) Performance and Myelin Heterogeneity Index Correlations between the myelin heterogeneity index in normal-appearing white matter and COWAT scores are shown for participants with multiple sclerosis (MS) and controls in 3 regions of interest. Lines denote lines of best fit.

Participants with MS had a mean score of 60 (range, 19-103) on the COWAT, whereas controls had a mean score of 69 (range, 55-88). Worse COWAT scores were correlated with increased MHI in NAWM of participants with MS in the SLF (*r* = −0.317; 95% CI, −0.549 to −0.078; *P* = .01) and cingulum (*r* = −0.335; 95% CI, −0.658 to −0.113; *P* = .006); however, the corpus callosum (*r* = −0.294; 95% CI, −0.535 to −0.053; *P* = .02) did not pass Bonferroni correction for multiple comparisons. No significant correlations were observed between MHI and COWAT scores in controls (Figure 3).

### Brief Visuospatial Memory Test–Revised

Participants with MS had a mean BVMT-R score of 49 (range, 1-71), whereas controls had a mean score of 56 (range, 25-68). Worse BVMT-R scores were correlated with increased MHI in NAWM of participants with MS in the SLF (*r* = −0.257; 95% CI, −0.582 to −0.011; *P* = .04), corpus callosum (*r* = −0.250; 95% CI, −0.505 to −0.002; *P* = .048), and cingulum (*r* = −0.266; 95% CI, −0.515 to −0.019; *P* = .04); however, these associations did not reach significance after Bonferroni correction. No significant correlations were observed between MHI and BVMT-R scores in controls (eFigure in the Supplement).

### MHI and Cognitive Performance

**Figure 4.**
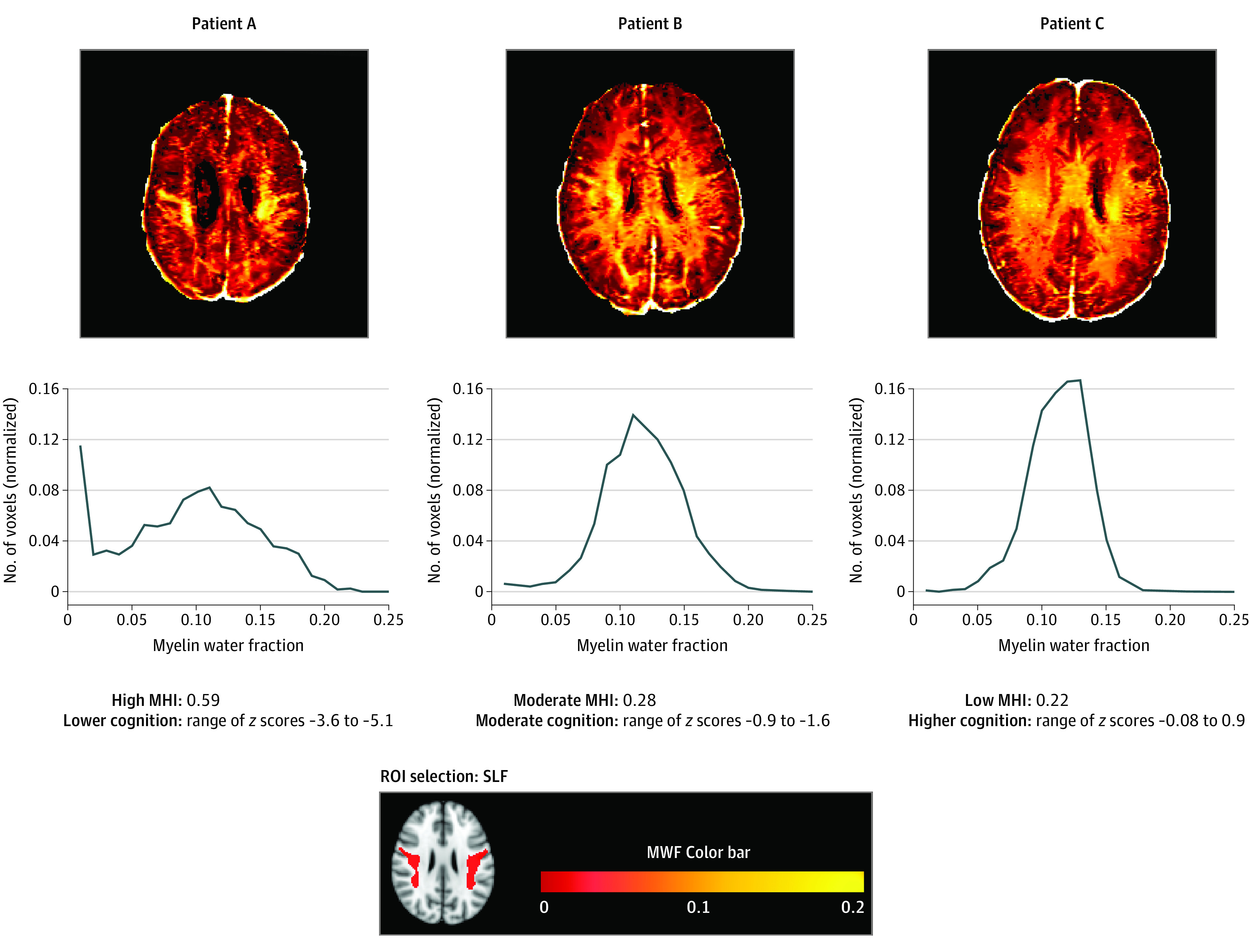
Axial Map of Myelin Water Fraction (MWF) Values, MWF Distributions in Superior Longitudinal Fasciculus (SLF), Myelin Heterogeneity Index (MHI) in SLF, and Cognitive *z* Scores in 3 Participants with Multiple Sclerosis Axial maps of MWF values (top), normalized histograms of MWF values in the SLF (middle), MHI in the SLF and cognitive *z* scores (bottom) of 3 participants with multiple sclerosis. Patient A had a high MHI in the SLF (0.59), matching their low cognitive scores compared with controls (range of *z* scores, −5.1 to −3.6). Patient B had both moderate MHI in the SLF (0.28) and cognitive test scores (range of *z* scores, −1.6 to −0.9). Patient C had a low MHI in the SLF (0.22) and performed at and above the level of controls on the cognitive tests range of *z* scores (−0.08 to 0.9). The *z* scores were calculated using the mean and SD for each cognitive test from the control sample. ROI indicates region of interest.

To illustrate the association between MHI and cognitive performance, Figure 4 depicts MWF maps and the distribution of MWF values within the SLF for high, moderate, and low MHI, and the associated range of cognitive *z* scores in 3 people with MS. The SLF was selected for this example as it most clearly exhibited an association with cognitive test scores in MS of the 3 ROIs. A range of cognitive *z* scores obtained from the 4 tests for each of the 3 patients are displayed as they are more informative than raw cognitive scores for this illustrative purpose. The *z* scores were calculated using the mean and SD for each cognitive test from our control sample. Patient A had a high MHI (0.59) in the SLF, matching their low cognitive scores compared with controls (*z* = −3.6 to −5.1). Patient B had both moderate MHI (0.28) in the SLF and cognitive test scores (*z* = −0.9 to −1.6). Patient C had a low MHI (0.22) in the SLF and performed at and above the level of controls on the cognitive tests (*z* = −0.08 to 0.9).

### Physical Disability (T25-FW and 9-HPT)

MHI was not associated with lower limb disability, as measured by the T25-FW, in corpus callosum (*r* = −0.018; *P* = .90), SLF (*r* = −0.007; *P* > .99), or the cingulum (*r* = 0.068; *P* = .60). In addition, MHI in the corpus callosum (*r* = 0.07; *P* = .60), SLF (*r* = 0.226; *P* = .08), and cingulum (*r* = 0.184; *P* = .20) was not associated with the 9-HPT.

## Discussion

Increased MHI in the MS cohort indicative of diffuse myelin abnormalities was found in NAWM of tracts that are associated with cognition. Furthermore, the MHI abnormalities correlated with cognitive deficits that are common in MS. Recent histopathological studies^15,16^ suggest that myelin in NAWM is affected in MS, and previous MWI reports^32^ suggested that decreased mean MWF and increased MWF heterogeneity (increased MWF variance in the brain) in NAWM is associated with worse physical disability. The MHI incorporates both MWF mean and variance; a decrease in MWF mean or an increase in MWF heterogeneity would result in an increased MHI. Thus, the MHI is sensitive to gross changes in MWF that shift the entire distribution, as well as smaller changes that broaden the distribution more subtly yet are clinically relevant.

Previous quantitative MRI studies using diffusion tensor imaging (DTI) or magnetization transfer imaging (MTI) have reported associations between abnormalities in NAWM and cognitive impairment in MS in the same brain regions as our study. DTI studies use fractional anisotropy (FA) as a general measure of microstructural WM tissue integrity. In WM, diffusion of water is restricted by microstructural components such as myelin. This causes the diffusion to be parallel, rather than perpendicular, to the direction of the axonal fibers, and it yields a high FA value in healthy WM. When microstructural damage to WM occurs, restrictions on the movement of water molecules are reduced, and diffusion becomes more isotropic. This results in a reduction in the FA.^33^ DTI also provides the mean diffusivity (MD) metric (equal to the magnitude of diffusion) with increased MD thought to represent microstructural abnormalities in WM.^34^ MTI studies typically report the magnetization transfer ratio, a semiquantitative metric that estimates the exchange of magnetization between nonaqueous tissue and water and is proposed to decrease with myelin loss.^35^

In the current study, we found a significant correlation between increased MHI in NAWM in the SLF, corpus callosum, and cingulum, and slower processing speed performance as measured by the SDMT. Similarly, decreased FA and magnetization transfer ratio,as well as increased MD, have been associated with worse processing speed in the SLF,^36,37,38^ corpus callosum,^36,37,38,39,40,41^ and cingulum NAWM.^36,37^ Likewise, we observed an association between increased MHI in NAWM in the SLF, corpus callosum, and cingulum, and decreased verbal memory (worse SRT scores). Associations between decreased FA in NAWM in these same 3 brain regions and impaired verbal memory have also been reported.^36^ In contrast to a previous study^36^ that failed to find significant associations between FA in NAWM and COWAT scores, we found significant correlations between MHI in NAWM and performance on the COWAT in participants with MS. This may be because MHI is a more specific measure of myelin damage than mean FA values. We noted the correlation between increased MHI in NAWM in SLF, corpus callosum, and cingulum, and decreased visuospatial memory as measured by the BVMT-R was *P* < 0.05, but it did not pass multiple testing correction. However, it is worth noting that increased FA and decreased MD in these brain regions and a significant association with decreased visuospatial memory has been observed in studies^36,37^ that did not use the conservative Bonferroni correction. It is possible that investigating visual pathway ROIs would lead to more significant correlations.

It was observed that there was a subset of patients with particularly low cognitive scores and high MHI values. Not surprisingly, these tended to be participants with progressive MS.^3^ This finding emphasizes the importance of including both relapsing-remitting and progressive phenotypes, with a broad spectrum of cognitive ability and severity of myelin damage representative of the population with MS as a whole, to comprehensively characterize the association between cognitive ability and severity of myelin damage in correlation studies.

To determine whether the observed associations between MHI values and cognitive performance were specific to cognition rather than a proxy for overall disability, we investigated the association between MHI in the 3 WM tracts we selected and performance on the T25-FW and the 9-HPT. We found no association between MHI in the corpus callosum, SLF, and cingulum and upper and lower limb disability. Therefore, we believe our findings are specific to cognitive function.

Although our results are consistent with previous DTI and MTI studies, MWI offers greater biological interpretability. DTI measures reflect a large number of biological changes that occur in MS, and caution is warranted when interpreting diffusion anisotropy changes as myelin changes.^42^ DTI measures are influenced by biological factors other than myelin, such as the directionality of fiber bundles, tortuosity, fiber crossings, and fiber orientation and coherence.^42,43,44,45,46^

Similarly, MTI estimates of macromolecular-bound water include but are not limited to myelin, and they are heavily influenced by inflammation and edema,^35^ which are often present in MS. In contrast, MWI has been validated with both human histological^18,19,47^ and animal models^20^ as a specific measure of myelin. We acknowledge that the denominator in the MWF is total water; therefore, increases in edema and inflammation can influence changes in MWF. However, if the MWF decreases in MS reported in the literature were due to only edema rather than myelin loss, dilution of the MWF from edema would require such significant swelling in the brain that it would result in a lethal increase in intracranial pressure.^14^ The use of MWI in the current study provides evidence that cognitive symptoms in MS are associated, at least in part, with myelin abnormalities in NAWM. This is of major clinical importance as it not only provides insight into the underlying pathology contributing to cognitive symptoms in MS, but also offers a noninvasive, tissue-specific biomarker for monitoring treatment efficacy, particularly for therapies geared toward remyelination.^48^

### Limitations

The study was limited to 1 hospital site, using a single scanner, which might restrict the generalizability of the results. However, the findings further support the use of MWI, and specifically MHI, to quantify MS-related demyelination and the association with cognitive impairment. This would be feasible for multicenter studies because MWI has excellent intersite^49^ and intervendor^50^ reproducibility.

## Conclusions

This study implements a myelin-specific imaging technique to demonstrate that otherwise normal-appearing brain tissue is diffusely damaged in participants wtih MS. We have also found that these changes are significantly associated with disease-related cognitive symptoms. These findings contribute to a better understanding of the underlying pathology involved in MS-related cognitive impairment; myelin damage in NAWM is likely playing a role. The MHI metric offers an in vivo marker feasible for use in clinical trials investigating cognitive symptoms in MS, for which a reliable, quantitative biomarker is sorely needed.

## Notes

Supplement. **eFigure.** Correlations Between BVMT-R Performance and MHI
