# Supplementary material for: Associations Between Findings From Myelin Water Imaging and Cognitive Performance Among Individuals With Multiple Sclerosis
Source: JAMA Netw Open. 2020 Sep 29;3(9):e2014220. doi: 10.1001/jamanetworkopen.2020.14220 (PMC7525360; doi:10.1001/jamanetworkopen.2020.14220)
Supplement: Supplement. — eFigure. Correlations Between BVMT-R Performance and MHI [file jamanetwopen-e2014220-s001.pdf]

Supplementary Online Content

Abel S, Vavasour I, Lee LE, et al. Associations between findings from myelin water imaging and cognitive performance among individuals with multiple sclerosis. *JAMA Netw Open*. 2020;3(9):e2014220. doi:10.1001/jamanetworkopen.2020.14220

eFigure. Correlations Between BVMT-R Performance and MHI

This supplementary material has been provided by the authors to give readers additional information about their work.

© 2020 Abel S et al. *JAMA Network Open.*

eFigure: Correlations Between BVMT-R Performance and MHI

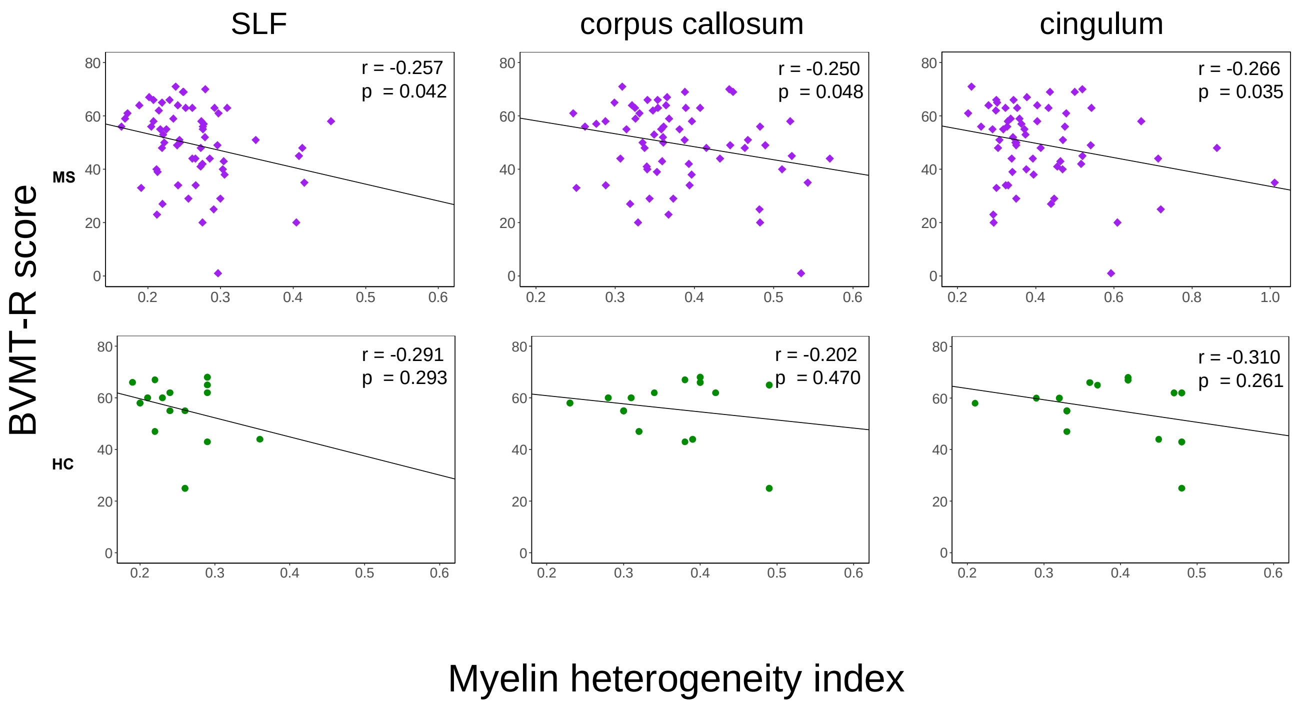

Correlations between the myelin heterogeneity index in NAWM (x axis) and BVMT-R scores (y axis) in MS (purple dots) and controls (green dots) in three ROIs. Lines = line of best fit. NAWM: normal appearing white matter; ROI: region of interest; MHI: Myelin heterogeneity index; BVMT-R: Brief Visual Spatial Memory Test – Revised; SLF: superior longitudinal fasciculus.

© 2020 Abel S et al. *JAMA Network Open.*
